# Transfer of *Westermannia difficilis* Dohrn to the genus *Polauchenia* McAtee & Malloch (Hemiptera, Heteroptera, Reduviidae, Emesinae, Emesini)

**DOI:** 10.3897/zookeys.1043.61344

**Published:** 2021-06-11

**Authors:** Hélcio R. Gil-Santana, Jürgen Deckert

**Affiliations:** 1 Laboratório de Diptera, Instituto Oswaldo Cruz, Av. Brasil, 4365, 21040-360, Rio de Janeiro, RJ, Brazil Instituto Oswaldo Cruz Rio de Janeiro Brazil; 2 Leibniz Institute for Evolution and Biodiversity Science, Museum für Naturkunde, Berlin, Germany Museum für Naturkunde Berlin Germany

**Keywords:** Assassin bugs, Colombia, Neotropical region, Venezuela

## Abstract

Based on the examination of its lectotype (here designated), *Westermannia difficilis* Dohrn, 1860 (Hemiptera, Heteroptera, Reduviidae, Emesinae, Emesini), currently included in *Dohrnemesa* Wygodzinsky, 1945, is transferred to the genus *Polauchenia* McAtee & Malloch, 1925 with the resulting new combination: *Polauchenia difficilis* (Dohrn, 1860), **comb. nov.** An updated key to the species of *Polauchenia* is provided.

## Introduction

There are about 30 genera of Emesinae classified in four tribes in the Neotropics (Wygodzinsky 1966; Maldonado 1990; Gil-Santana et al. 2015; Castro-Huertas et al. 2020).

When describing *Westermannia difficilis*, Dohrn (1860) provided a very short description of the species, apparently based on a single specimen from Colombia and deposited in “Berliner Museum”, but he did not mention the gender of the specimen. Dohrn (1863) presented a redescription of *W. difficilis*, including more details of its coloration and stated that this species was based on one specimen deposited in the Berliner Museum, but again, its gender was not mentioned. Champion (1899) mentioned a “single specimen” of *W. difficilis* Dohrn, 1860, from Panama, which was thought to probably belong to *Emesa* Fabricius, 1803 by Wygodzinsky (1945), who later (Wygodzinsky 1966) stated that judging by Champion’s figure, it probably represented another species of the “*difficilis* group” of *Dohrnemesa* Wygodzinsky, 1945.

Because *Westermannia* Dohrn, 1860 was preoccupied by a Hübner’s genus of the same name in Lepidoptera ([1821]) (McAtee and Malloch 1925), Kirkaldy (1904) proposed a new name, *Westermannias*, to replace it. McAtee and Malloch (1925) synonymized *Westermannia* and *Westermannias* with *Emesa*, including “*difficilis* (*Westermannia*) Dohrn” and “*tenerrima* (*Westermannia*) Dohrn” as unplaced species in the genus *Emesa*. McAtee and Malloch (1925) argued that they were unable to place these species in their keys without a fuller knowledge of the characters of their types.

When describing *Dohrnemesa*, Wygodzinsky (1945) commented about the difficulties in establishing the systematic position of *Westermannia difficilis* because the types were not examined again. However, he considered that, considering the generic synonyms of *Westermannia* and *Westermannias* with *Emesa* proposed by McAtee and Malloch (1925), there was no reason to worry too much with the species previously included in these genera, because they might eventually be included in *Dohrnemesa* in the future.

Wygodzinsky (1945) considered *Dohrnemesa* close to *Polauchenia* McAtee & Malloch, 1925 and pointed out that the main differences between them were the absence of tubercles or spined humeri and the presence of a free short vein emitted from the base of the basal cell of the forewing in *Dohrnemesa*. In the key to genera of the Emesini presented by Wygodzinsky (1966), the presence or absence of a short free vein at the base of the basal cell of the forewing, in *Dohrnemesa* and *Polauchenia*, respectively, is the main character that separates these genera. Additionally, based on this characteristic alone, Wygodzinsky (1966) transferred *Polauchenia reimoseri* Wygodzinsky, 1950 to *Dohrnemesa*, assuming that the possession of two veins emitted from the base of the basal cell indicated that this species belonged to the latter genus (Gil-Santana and Ferreira 2017). The new combination, *Dohrnemesa difficilis* was also established by Wygodzinsky (1966), although he did not mention his reasons to include this species in *Dohrnemesa*. On the other hand, he divided *Dohrnemesa* in two groups, claiming that the species of the “*difficilis* group” would have the fore lobe subglobular, abruptly narrowed behind and distinctly separated from petiole, and a strongly widened abdomen with flaring connexival segments.

In the current work, we confirm that the lectotype (here designated) of *Westermannia difficilis* Dohrn, 1860, a species currently included in *Dohrnemesa* Wygodzinsky, 1945, does not belong to this genus but to *Polauchenia* McAtee & Malloch, 1925, resulting in the new combination: *Polauchenia difficilis* (Dohrn, 1860), comb. nov. The main differences are that in *P. difficilis*, comb. nov. the base of basal cell is pointed, emitting a single longitudinal vein, with the absence of a short free vein at this base and the humeri are spined. Both differences are sufficient to show that *P. difficilis*, comb. nov. does not belong to *Dohrnemesa* but to *Polauchenia*. Additionally, as commented below, several other diagnostic characteristics of *Polauchenia* are present in the specimen examined.

## Material and methods

For the present study, the male lectotype (here designated) of *Westermannia difficilis* Dohrn, deposited in the Hemimetabola Collection of the Museum für Naturkunde Berlin, Leibniz Institute for Evolution and Biodiversity Science, Berlin, Germany (MFNB), was directly examined (Figs 1–2, 4–14).

The photos of the lectotype (here designated) of *W. difficilis* Dohrn, 1860 were taken with a Canon EOS 6D (Fig. 1) and M50 (Figs 2–12, 14) with a MP-E 65 mm f/2.8 1–5× macro lens attached. Multiple focal planes were merged using the auto-montage software Helicon Focus Pro.

Figure 13 was produced by drawing the outline of the margins and veins of the forewing directly from its photograph (Fig. 12), using CorelDRAW Graphics Suite 2020.

General morphological terminology mainly follows Wygodzinsky (1966). However, the [visible] segments of the labium are numbered as II to IV, given that the first segment is lost or fused to the head capsule in Reduviidae (Weirauch 2008; Schuh et al. 2009).

When describing label data, a slash (/) separates the lines and a double slash (//) different labels.

## Results

### Taxonomy

**Subfamily Emesinae**

#### Tribe Emesini

##### Polauchenia difficilis

Taxon classificationAnimaliaHemipteraReduviidae

(Dohrn, 1860) comb. nov.

E0BAD3D1-3D24-55C7-9A60-A76373FABAE8

Figs 1 , 2 , 4–7 , 8–11 , 12–14

Westermannia difficilis Dohrn, 1860: 251 [description], 1863: 47–48 [redescription]; Stål 1872: 125 [checklist]; Walker 1873: 150 [catalog]; Lethierry and Severin 1896: 71 [catalog]; Champion 1899: 164, pl. 10, figs 8, 8a [record of a supposed specimen from Panama]; McAtee and Malloch 1925: 46–47 [as “unplaced species” listed among species of Emesa]; Wygodzinsky 1945: 252 [discussion about the future possibility of its placement in Dohrnemesa], 1949: 34 [catalog, as Emesinae*incertae sedis*].
Dohrnemesa difficilis ; Wygodzinsky 1966: 231, 237 [citation, key; checklist, statement that W. difficilis figured by Champion 1899 from Panama is not the same species]; Gil-Santana and Ferreira 2016: 584 [citation], 2017: 203, 229 [citation, key].

###### Type material examined.

*Westermannia difficilis*, male lectotype (here designated): [handwritten label]: *Leptol*. / *difficilis* / Dohrn // [blue underlined handwritten label]: Columb; Moritz. // [printed label]: 3326 // [printed label]: [at right side]: QR CODE, [at left side]: http://coll.mfn-berlin.de/u/ /123b88 // [printed red label]: LECTOTYPE / *Westermannia difficilis* Dohrn, 1860 / designated by H. R. Gil-Santana & / J. Deckert 2020 (MFNB).

###### Notes.

In the old catalogue of the Berliner Museum the specimen examined here was registered under the number 3326 and named as *Leptolemus difficilis* Dohrn (Fig. 3). The name of the genus “*Leptolemus*” [apparently abbreviated as “*Leptol*.”] can also be read on the label attached to the type specimen (Fig. 2). In the same catalogue, other species, *Leptolemus tenerrima* was also listed just above *L. difficilis* (Fig. 3). However, when describing these two species, Dohrn (1860) included them in *Westermannia*, described in the same occasion too. As far as it seems, the name *Leptolemus* is a manuscript name which was never applied as a published name to any Emesinae or other Reduviidae (e.g., Lethierry and Severin 1896; Wygodzinsky 1966; Maldonado 1990).

The collector of the specimen, Moritz (Johann Wilhelm Karl Moritz 1797–1866), collected in the Caribbean islands and Venezuela, but there is a disagreement among some authors if he collected in Colombia. It is possible that the records of his collecting from Colombia originated from the confusion between Venezuela and Colombia, parts of the former having once belonged to the ancient vice-kingdom of “Nueva Granada” (Papavero 1973). In this case, there is a possibility that the lectotype (here designated) of *W. difficilis* was collected in Venezuela and not in Colombia as stated in his collecting data.

###### Diagnosis.

*Polauchenia difficilis*, comb. nov. can be separated from other species of the genus by the combination of characters presented in the key below. *Polauchenia difficilis*, comb. nov. shares similarities with *P. paraprotentor* Gil-Santana & Ferreira, 2017 but differs from this species in several characteristics, such as: the pale markings of the antenna, middle and hind femora are simple (*P. paraprotentor*) or bordered by darker markings (*P. difficilis*, comb. nov.), those on antenna narrow, with the pale annuli as long or only slightly longer (*P. paraprotentor*) or quite longer (four and seven times) than the width of the segment (*P. difficilis*, comb. nov.); fore coxa with a median pale annulus (*P. difficilis*, comb. nov.) or two pale annuli at submedian basal portion and approximately midportion of distal half of the segment (*P. paraprotentor*); distal portion of forewings with (*P. difficilis*, comb. nov.) or without (*P. paraprotentor*) a large whitish subdistal marking; petiole approximately 1.5 (*P. paraprotentor*) or 1.3 (*P. difficilis*, comb. nov.) times as long as fore lobe; humeri spined (*P. difficilis*, comb. nov.) or not (*P. paraprotentor*); spine of scutellum obliquely directed upwards (*P. difficilis*, comb. nov.) or backwards (*P. paraprotentor*); spines of scutellum and metanotum mostly pale (*P. difficilis*, comb. nov.) or brownish (*P. paraprotentor*).

**Figures 1–3.**
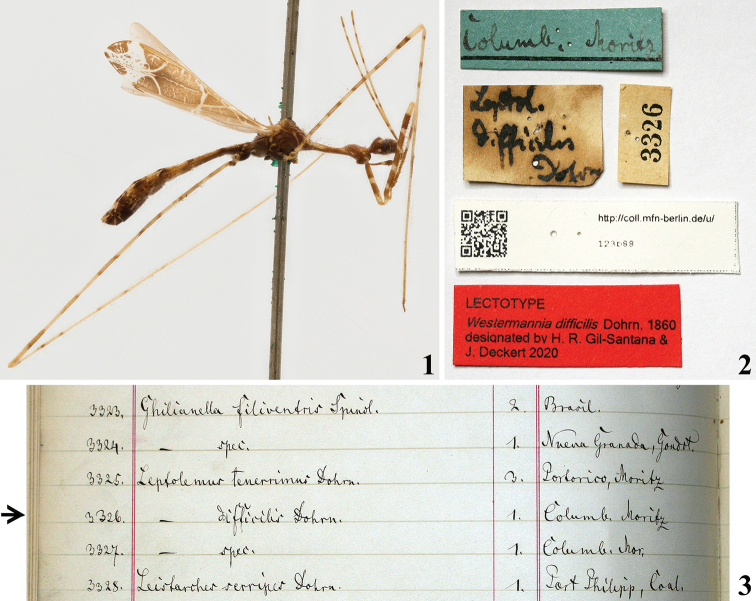
**1, 2***Polauchenia difficilis* (Dohrn, 1860), comb. nov., male lectotype (here designated) **1** lateral view, right side **2** labels **3** old catalogue of the Berliner Museum, page excerpt, arrow points to the record of *Leptolemus difficilis* Dohrn in it.

###### Redescription.

**Figures 4–7.**
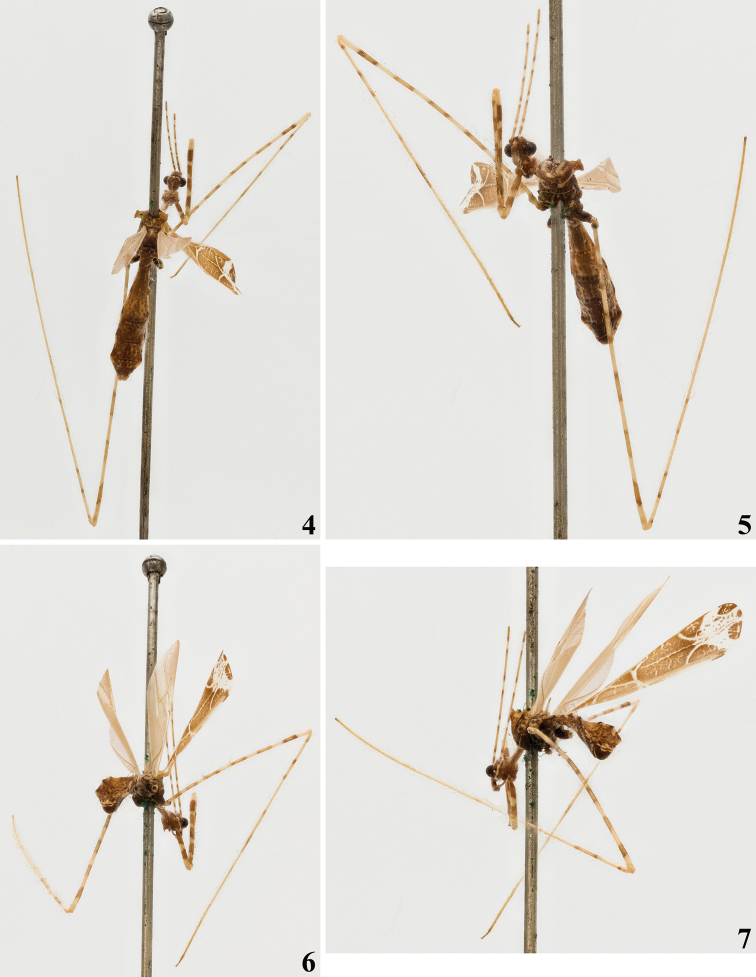
*Polauchenia difficilis* (Dohrn, 1860), comb. nov., male lectotype (here designated) **4** dorsoposterior view **5** anteroventral view **6, 7** ventroposterior views.

**Figures 8–11.**
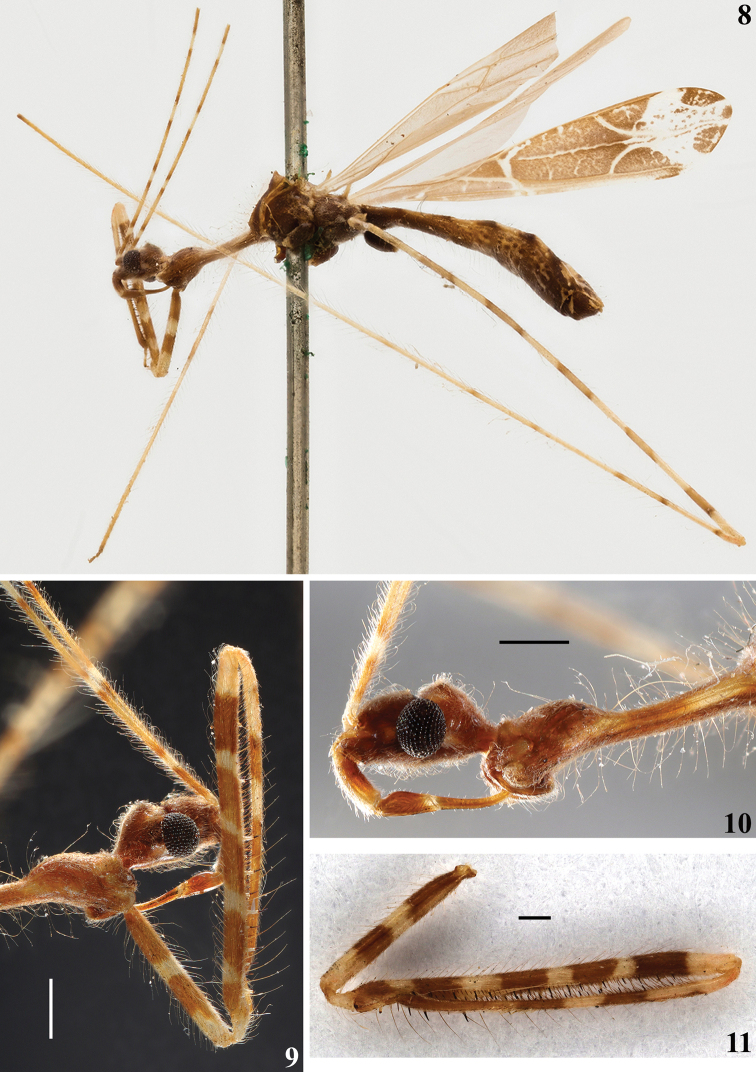
*Polauchenia difficilis* (Dohrn, 1860), comb. nov., male lectotype (here designated) **8** ventrolateral view, left side **9** fore lobe of pronotum, head and right fore leg, lateral view **10** head, fore lobe and petiole of pronotum, lateral view **11** left fore leg detached from the specimen, lateral view. Scale bars: 0.5 mm (**9, 10**); 0.26 mm (**11**).

**Figures 12–14.**
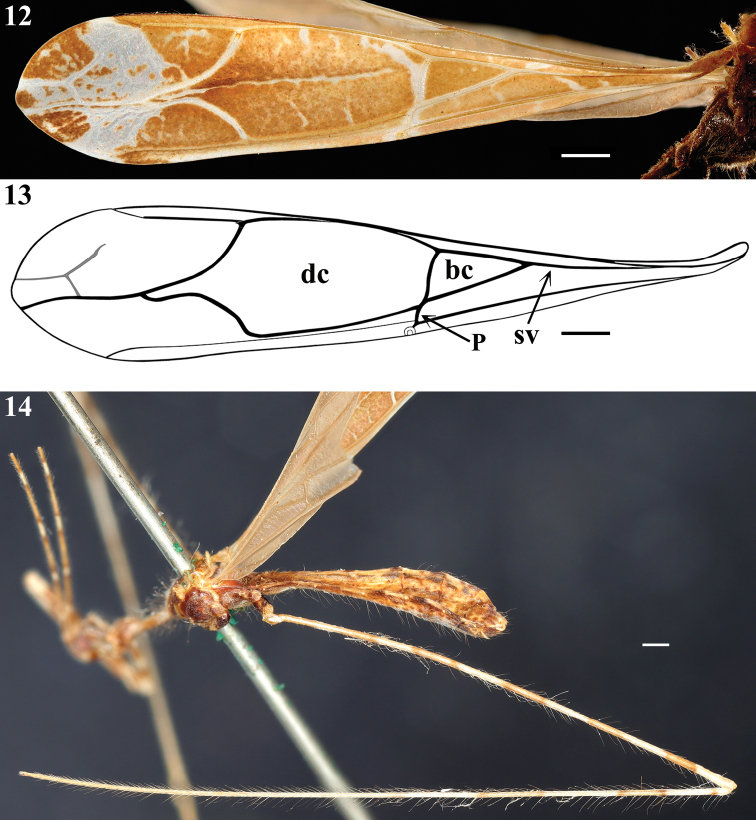
*Polauchenia difficilis* (Dohrn, 1860), comb. nov., male lectotype (here designated) **12, 13** forewing **13** schematic outline **14** meso and metathorax, abdomen and left hind leg, lateral view. Abbreviations: bc basal cell, dc discal cell, P Pcu cross vein, sv single vein emitted from the basal cell. Scale bars: 0.5 mm (**12–14**).

**Male.** Measurements (mm): total length: to tip of abdomen 10.0; to tip of forewings 10.6. *Coloration*: brownish to light brown, with yellowish to pale markings or portions (Figs 1, 4–12, 14). Head brownish; clypeus and labrum paler; pale small spots around the eye: in front of the midpoint of its anterior margin, and a pair behind its posterior margin, above and below the level of the anterior spot; the former slightly larger and just behind transverse sulcus; a pale whitish median small area behind transverse sulcus; a median longitudinal dorsal narrow pale whitish stripe on posterior half of postocular region; apices of labial segments II and III and base of labial segment IV pale white to pale yellow; segment IV somewhat paler (Figs 1, 4, 5, 8–10). First antennal segments (others absent) pale brownish with basal portion and four pale annuli; the latter bordered by contiguous basal and distal darkened annuli, forming a set of bicolored annuli; the two distal set of annuli with the pale portion somewhat larger than the two basal ones; the pale portion of the annuli approximately four and seven times the width of the segment in the basal and distal annuli, respectively while the darkened basal and distal annuli are narrower, slightly longer to twice longer the width of the segment, respectively (Figs 1, 4, 5, 8, 9). Thorax: brownish, anterior collar paler; prothoracic supracoxal lobes pale whitish on its anterior margin; a rounded pale spot above the latter; petiole paler laterally, on the portion just behind the fore lobe and more extensively on distal portion (Figs 1, 8–10). Hind lobe of pronotum: a pair of contiguous pale whitish longitudinal stripes on anterior portion, the medial (submedian) stripes approximately half longer than the lateral ones, running approximately on the basal third of the hind lobe; lower margin pale at approximately its anterior two-thirds; humeral tubercles, including their spines pale (Figs 1, 5, 8). Spines of scutellum and metanotum pale, with their tips somewhat darkened (Figs 1, 4, 7, 8, 14). Meso- and metapleura generally dark brownish; meso- and metathoracic supracoxal lobes pale whitish on their posterior margin (Figs 1, 8, 14). Legs: fore coxa light brownish with a median annulus and approximately the apical fourth pale whitish; middle and hind coxae brownish with their distal margin somewhat paler; fore trochanter with approximately basal half pale and distal half brownish; middle and hind trochanters pale with an ill-defined median brownish spot (Figs 1, 8, 9, 11). General coloration of fore femora brownish, with four narrow annuli and apex, more extensively, pale; larger spiniferous processes with their basis whitish and the distal spine blackish (Figs 1, 9, 11). Middle and hind femora generally pale with six large dark annuli, which are bordered by contiguous narrower darker annuli, forming a set of bicolored large annuli, the first at base of the femora, somewhat smaller, the following ones separated by a distance approximately equivalent to the total length of each annulus (including their darker extremities), the more distal, far from apex by approximately the same equivalent distance (Figs 1, 4–6, 8, 14). Fore tibia mostly pale brownish with approximately the basal fourth and a submedian basal annulus pale; on the basal fourth, a small pair of dark spots on dorsal and ventral surfaces approximately at midpoint of this pale portion (Figs 1, 9, 11). Middle and hind tibiae pale, a subbasal, small, dark spot on dorsal surface followed by two large faintly dark annuli, bordered by contiguous, narrow, darker annuli, the more distal somewhat larger, after them, a small, dark annulus approximately as far as the distance between the previous large annuli; all these markings on the basal half and basal third of the segment of middle and hind tibia, respectively; apices of both tibiae darkened towards apices (Figs 1, 4–8, 14). Fore tarsi pale brownish, second tarsomere paler (Fig. 11); middle and hind tarsi absent. Forewing brownish with most veins paler to whitish; a few oblique ill-defined, small, pale stripes or markings on basal half, between veins; a large, curved, whitish stripe running over Pcu cross vein, distal vein of basal cell and basal portion of discal cell; a diffuse texture formed by small, irregular, whitish spots or transverse lines inside discal cell and a narrow, longitudinal, submedian, oblique, somewhat irregular line along discal cell, except at its basal portion, and two larger, oblique, whitish stripes over distal veins of the discal cell, which join a large whitish spot, which runs transversely obliquely towards apex, subdistally between lateral margins of the wing and medially attaining the apex of the wing at its median portion; this large whitish marking is speckled with brownish spots at median portion; the lateral portions to this large whitish marking are otherwise brownish and speckled by several whitish markings; the tip of the wing is shortly brownish at its median portion (Figs 1, 8, 12). Hind wings hyaline; veins somewhat darker (Figs 6–8). Abdomen generally brownish with scattered, ill-defined, pale and dark markings and spots, respectively; connexivum pale with distal dark spots, which are proportionally larger in relation to the pale basal portion on the last three segments; sternites additionally with thin, interrupted and ill-defined pale lines; posterolateral margins of sternite VIII pale whitish; genital capsule dark with parameres pale (Figs 1, 4–8, 14). *Vestiture*: integument covered with very numerous and long thin setae, and with a short and very dense pubescence formed by thin, curved or adpressed setae (Figs 1, 8–11, 14). Fore femur: posteroventral series beginning at the base of the article and ending far from apex, composed of about 11 large and medium-sized spiniferous processes, the most basal of which with its apex slightly inclined toward apex of article. A sparse series of very long, darker and strong setae accompanies the posteroventral series. Lengths of larger processes combined with apical spines about as long as or somewhat shorter than the diameter of segment. Fore tibiae with numerous stiff setae on subapical dorsal depression (Figs 9, 11). Forewing almost completely glabrous, with a few scattered short thin setae at basal portion and a few scattered somewhat longer ones along costal vein (Fig. 12). Hind wings glabrous. *Structure*. Integument moderately shiny. Head (Figs 1, 4, 5, 8–10): elongated; anteocular portion longer than postocular. Transversal (interocular) sulcus deep, situated somewhat anteriorly to middle of eyes. Eyes globose, reaching dorsal outline of head at interocular sulcus and not reaching ventral outline of head; a pair of very short tubercles just behind interocular sulcus. Antenna inserted closer to apex of head than to the eyes; first antennal segment thin, slender; others absent. First two visible labial segments thicker than the distal segment; apex of segment III slightly posteriorly to level of midportion of eye; segment IV ending close to midpoint of stridulitrum at its anterior portion. Thorax (Figs 1, 4–14): pronotum pedunculate; petiole approximately 1.3 times as long as fore lobe, the latter semioval; humeral rounded tubercle with a short acute spine. Spines of scutellum and metanotum somewhat elongated, obliquely directed upwards, apices acute, the latter somewhat longer than the former. Fore legs slender; fore coxae elongated, approximately 1.3 times longer than petiole; fore tibiae thinner and slightly shorter than fore femora, somewhat curved; slightly depressed in dorsal portion subapically; somewhat thickened at apex. Mid and hind legs very long, slender, slightly curved; tibiae somewhat thinner and longer than femora. Fore tarsus short, three-segmented, slender; other absent. Forewings slender; basal cell triangular, with a single directed vein emitted from its base and with Pcu cross vein meeting it slightly posterior to the level of its apical portion; pterostigma ending somewhat far from apex of the wing. Abdomen: slender, slightly enlarged towards posterior half. Last tergite narrowed towards apex, subtriangular, posterior margin rounded, with a short prolongation posteriorly, covering most of the genital capsule. Eighth sternite covering approximately half of the pygophore, ventrally.

## Discussion

The transfer of *D. difficilis* to *Polauchenia* is in accordance with the aforementioned assertion that *Dohrnemesa* can be separated from *Polauchenia* by the absence/presence of spined humeri and the presence/absence of a free short vein emitted from the base of the basal cell of the forewing in the former and latter genus, respectively (Wygodzinsky 1966; Gil-Santana and Ferreira 2017).

Yet, the inclusion of *P. difficilis*, comb. nov. in *Polauchenia* agrees with the following diagnostic features of the genus (McAtee and Malloch 1925; Wygodzinsky 1966; Forero 2004): medium-sized species (11–20 mm in length); pronotum pedunculate (Figs 1, 8, 10); scutellum and metanotum with a spine (Figs 1, 8); posteroventral series of fore femora beginning at base of article, composed of large and small spiniferous processes bearing relatively slender apical spines; large processes of subequal size, the most basal either straight or slightly inclined toward apex of article; fore tarsi three-segmented (Figs 9, 11); forewings with two cells, base of basal cell pointed, emitting a single longitudinal vein towards axillary region (Figs 1, 12, 13).

Additionally, among other characteristics of *Polauchenia*, the following are noteworthy and also present in *P. difficilis*, comb. nov.: all species are conspicuously marked with light and dark colors (e.g., Figs 1, 8–12); petiole of pronotum ranging from slightly shorter to much longer than fore lobe of pronotum (Figs 1, 8, 10) and series of processes of the fore femora often accompanied by strong elongated setae (Figs 9, 11) (Wygodzinsky 1966; Gil-Santana and Ferreira 2017).

The only small difference is the total length, which was recorded as being 10.6 mm for *P. difficilis*, comb. nov., very close to the minimum stated by previous authors (11 mm), allowing us to state 10.6 mm as the actual minimum of the genus. Moreover, it is noteworthy that Dohrn (1860, 1863) recorded 11 mm as the length of the lectotype of *W. difficilis*. It is not possible to know how accurately A. Dohrn measured the specimen or if he rounded the measurement to an exact number. It is possible that the specimen was originally 11 mm in length when examined by him but due to the passing of time, the specimen may have shortened a little.

In any case, it becomes clear that the transfer of the species studied here from *Dohrnemesa*, the genus where it was currently included (Wygodzinsky 1966) to *Polauchenia* is in accordance with the differences between these genera and the diagnostic characteristics of *Polauchenia*.

On the other hand, some characteristics which Wygodzinsky (1966) believed *P. difficilis*, comb. nov. would have (fore lobe subglobular, abruptly narrowed behind and distinctly separated from petiole; a strongly widened abdomen with flaring connexival segments) are absent in this species. Therefore, it becomes clear that Wygodzinsky (1966) had limited knowledge of the species and certainly never examined the type of *W. difficilis*, providing an additional argument to disregard his placement of *P. difficilis*, comb. nov. in *Dohrnemesa*.

The type specimen of *Westermannia difficilis* was designated here as a lectotype following the Art. 74.1 of ICZN.

Taking into account the taxonomical change proposed here, seven species are now included in *Polauchenia* and nine in *Dohrnemesa* (*D. albuquerquei* Wygodzinsky, 1966, *D. buyassuana* Wygodzinsky, 1958, *D. carvalhoi* Wygodzinsky, 1966, *D. exporrecta* Wygodzinsky, 1958, *D. kuarajucassaba* Gil-Santana & Ferreira, 2017, *D. lanei* Wygodzinsky, 1945, *D. oliveirai* Gil-Santana & Ferreira, 2016, *D. reimoseri* (Wygodzinsky, 1950), *D. santosi* Wygodzinsky, 1945) (Wygodzinsky 1966; Gil-Santana and Ferreira 2016, 2017).

### Key for the species of *Polauchenia*, modified from Wygodzinsky (1966) and Gil-Santana and Ferreira (2017)

**Table:** 

1	Postocular region of the head with a median spine, besides a pair of lateral spined tubercles	***unicornis* Maldonado, 1968**
–	Postocular region of the head without a median spine, with or without a pair of lateral spined or rounded tubercles	**2**
2	Petiole of pronotum quite longer, at least 1.3 times as long as the fore lobe	**3**
–	Petiole of pronotum little, if any longer than fore lobe	**6**
3	Petiole of pronotum approximately 1.3–1.5 times as long as fore lobe; length 10.6–14 mm	**4**
–	Petiole of pronotum at least twice longer than the length of fore lobe; length 15 mm or longer	**5**
4	Petiole of pronotum approximately 1.5 times as long as the fore lobe; length 14 mm; pale markings of the antenna, middle and hind femora simple	***paraprotentor* Gil-Santana & Ferreira, 2017**
–	Petiole of pronotum approximately 1.3 times as long as the fore lobe; length 10.6 mm; pale markings of the antenna, middle and hind femora bordered by darker markings	***difficilis* (Dohrn, 1860)**
5	Length 17.5 mm; females (males unknown) brachypterous, the forewing reaching at about middle of abdomen	***marcapata* Wygodzinsky, 1966**
–	Length not more than 15 mm; female slightly brachypterous, forewing reaching far posterior to the middle of abdomen	***protentor* McAtee & Malloch, 1925**
6	Length 11 mm; postocular region of the head without projections, spines of scutellum and metanotum yellowish	***schubarti* Wygodzinsky, 1950**
–	Length 16 mm; postocular region of the head with a pair of spined conical tubercles; spines of scutellum blackish	***biannulata* McAtee & Malloch, 1925**

## Supplementary Material

XML Treatment for Polauchenia difficilis
